# Views of College Students on Plastic Surgery

**Published:** 2013-06

**Authors:** Muhammad Ahmad, Humayun Mohmand, Nabila Ahmad

**Affiliations:** 1 Plastic, Reconstructive, Hand and Hair Restorative Surgeon, La Chirurgie, Islamabad Cosmetic Surgery Centre, Islamabad, Pakistan;; 2 Cosmetic Plastic and Hair Restorative Surgeon; La Chirurgie, Islamabad Cosmetic Surgery Centre, Islamabad, Pakistan;; 3 Foundation University, Islamabad, Pakistan

**Keywords:** Views, College, Student, Plastic surgery

## Abstract

**BACKGROUND:**

Various studies have been conducted in many countries to determine the perception/awareness about plastic surgery. The present study assessed the views of college students about plastic surgery.

**METHODS:**

A questionnaire consisted of nine questions regarding the basic knowledge about plastic surgery was randomly distributed among college students. The students were given 20 minutes to fill out the forms.

**RESULTS:**

A total of 250 male and 250 female college students were randomly included in the study. The mean age of the male students was 21.1 years as compared to 20.7 years of female students. The top five conditions named were related to hair (89.8%) followed by face scars (88%). The most common procedure named by the students was liposuction (88.2%) followed by hair transplantation. 80.2% of the students opted not to be a plastic surgeon if given an opportunity to select the profession. 33.8% of the students had seen some kinds of plastic surgery operation. Only 5.6% of the students (3.4% male and 2.2% female) had seen some kinds of plastic surgery procedure. 68% of male students and 48% of female students wished to have a plastic surgery procedure sometime in their lives. Majority of the students (88%) got the information from the internet. The second most common source was magazines (85.2%). Majority of the students (53.4%) had an idea of an invisible scar as a result of having a plastic surgery procedure. Only 22% thought to have no scar. Late Michael Jackson was at the top of the list of celebrities having a plastic surgery procedure (97.8%) followed by Nawaz Shariff (92.4%).

**CONCLUSION:**

Despite the rapid growth of plastic surgery in the last two decades, a large portion of population remains unaware of the spatiality. It is essential to institute programs to educate healthcare consumers and providers about the plastic surgery.

## INTRODUCTION

The specialty of plastic surgery is not very new. Scripts have been found in the ancient Papyrus. The procedure for nasal reconstruction was developed by Sushruta, an Indian surgeon. The development of plastic surgery as a specialty has resulted in a significant positive impact on health care, and the function and expertise of the plastic surgeons are well-defined, however, they are poorly known in the society. In spite of their extensive surgical training and technical skills, plastic surgeons are often recognized as performing only cosmetic surgeries.^1^ This all is because of media and celebrity worship. Media plays an important role in public education and opinion making. In Europe and America, both men and women are becoming increasingly concerned about their physical appearance and are seeking cosmetic enhancement, and most studies report that people are generally happy with the outcome of cosmetic procedures.^2^ According to American Society of Plastic Surgeons, nearly 12.1 million cosmetic procedures were performed in 2008.^3^ Similarly in another study among Norwegian women between the ages of 22 and 55 years, 7.7% reported that they had undergone cosmetic surgery, while 22.6% indicated a wish for such a procedure.^4^

Various studies have been conducted in many countries to determine the perception/awareness about plastic surgery.^1^^,^^5^^–^^7^ The present study is of the first of its type in Pakistan assessing the perception of college students about plastic surgery. 

## MATERIALS AND METHODS

**Fig. 1:**
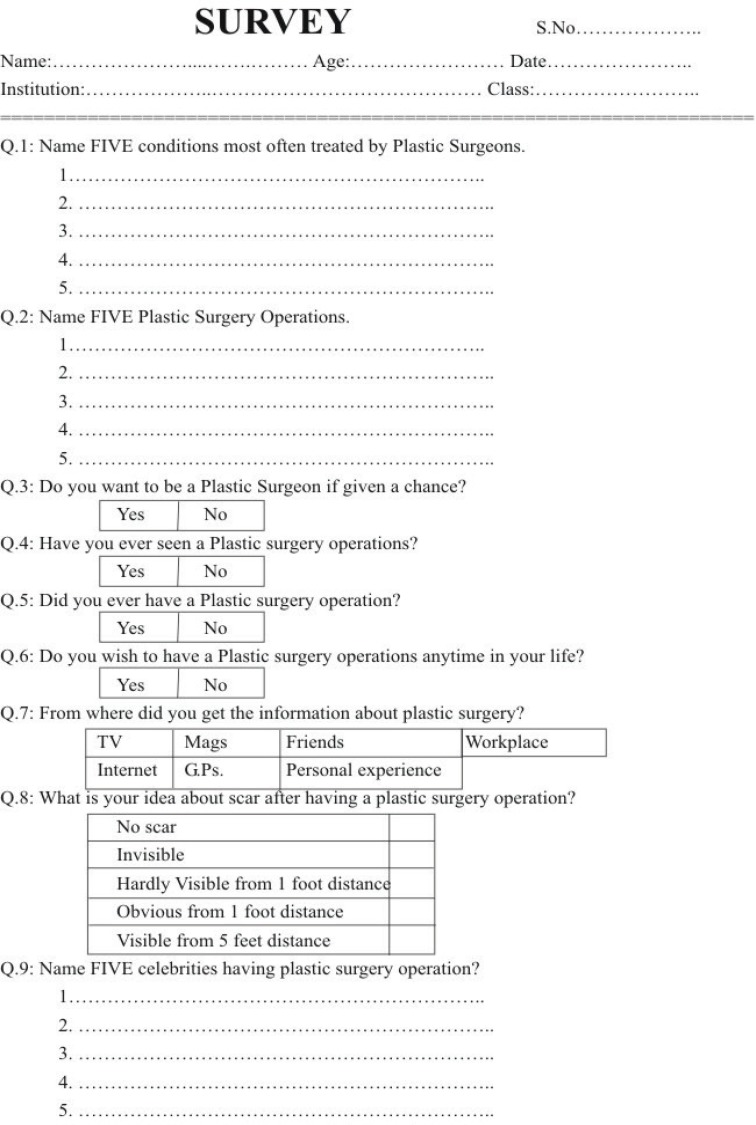
Questionnaire.

A questionnaire based survey was conducted among male and female college students. The professional colleges of education, medical and engineering colleges were included. The information sought in the questionnaire included age and gender of the respondents. The questionnaire consisted of nine questions regarding the basic knowledge about plastic surgery (Figure 1). The colleges and students were all randomly selected. From each college, 50 students were selected. Four single-sex colleges (two male and two female) were enrolled and two co-education colleges were randomly chosen. The personal information about the students was kept confidential. The questionnaire was distributed in the classrooms and the students were allowed for 20 minutes to fill out the forms and they were collected on the same day. The questionnaires were distributed on different days in different colleges. The questionnaires were collected in the presence of principle investigator. All the collected data was tabulated and analysed using Microsoft ^®^ Excel ^®^ and SPSS (version 10.0, SPSS Inc., Chicago, USA).

## RESULTS

A total of 250 male and 250 female college students were randomly included in the study. Two male and two female colleges were enrolled with 50 students from each college. Similarly 50 male and 50 female students were selected from three co-education colleges. The mean age of the male students was 21.1 years as compared to 20.7 years of female students.

**Fig. 2:**
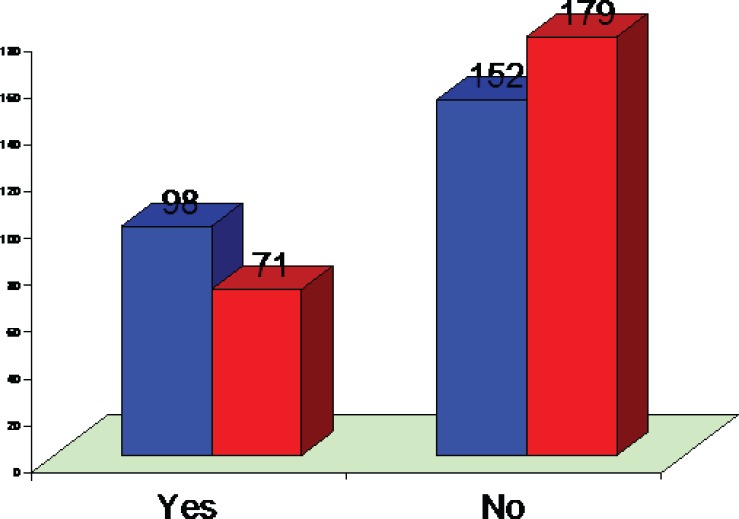
Plastic surgery procedure observed

**Fig. 3:**
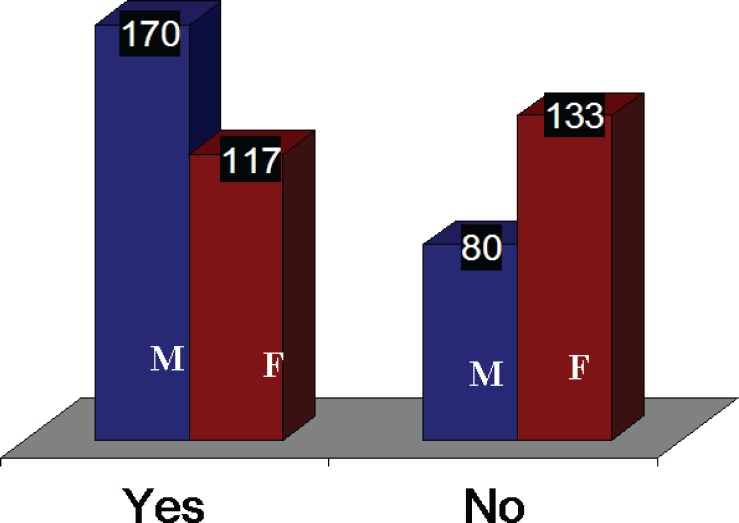
Wish to have a plastic surgery procedure

**Fig. 4:**
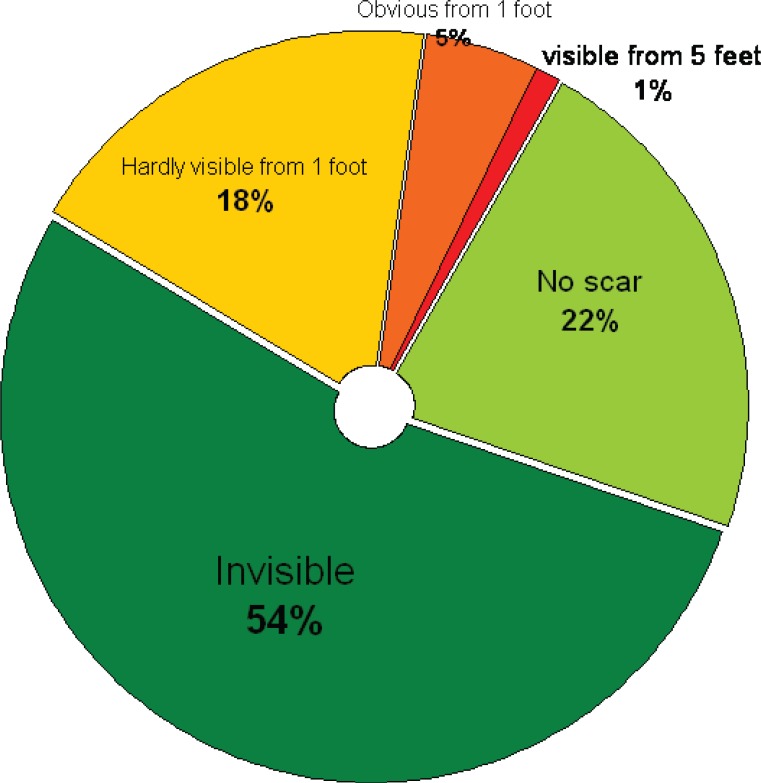
Ideas about scar after plastic surgery

The top five treated conditions were related to hair (89.8%) and face scars (88%) (Table 1). The most common top 5 plastic surgery operations reported by the students were liposuction (88.2%) and hair transplantation (84.4%) (Table 2). Regarding the future of plastic surgery, 80.2% of the students opted not to be a plastic surgeon if given an opportunity to select the profession. 33.8% of the students reported previous observation of some kinds of plastic surgery (Figure 2). Only 5.6% of the students (3.2% male and 2.4% female) had undergone some kinds of plastic surgery and reported an experience. 68 % of male students and 47% of female students wished to have a plastic surgery procedure sometime in their lives (Figure 3). Regarding source of information, the majority of the students (88%) reported to get the information from the internet. The second most common source was magazines (85.2%) (Table 3). The majority of the students (53.4%) had an idea of an invisible scar as a result of having a plastic surgery procedure and just 22% did not mention any scar (Figure 4) and finally the top 5 celebrities were Late Michael Jackson as at the first celebrity having a plastic surgery (97.8%) followed by Nawaz Shariff (92.4%) (Table 4).

**Table 1:** Five conditions treated by Plastic Surgeons (n=500).

**Related conditions **	**Total respondents**	**%**
Hair	449	89.8
Face scars	440	88.0
Nose	417	83.4
Face trauma	415	83.0
Scars	412	82.4
Moles	376	75.2
Burns	367	73.4
Wounds	300	60.0
Aging skin	288	57.6

**Table 2:** Five Plastic Surgery Operations (n=500).

**Operation**	**Total respondents**	**%**
Liposuction	441	88.2
Hair transplant surgery	422	84.4
Nose reshaping	363	72.6
Skin grafting	358	71.6
Laser	357	71.4
Cleft lip	350	70.0
Breast surgery	305	61.0
Skin rejuvenation	282	56.4

**Table 3:** Source of Information

**Media**	**Male **	**Female **	**%**
Internet	210	230	88.0
Magazines	231	195	85.2
Friends	189	125	62.8
Newspapers	112	170	56.4
Bill boards	92	85	35.4
TV	95	76	34.2
General practitioners	10	3	2.6

**Table 4:** Celebrities with plastic surgery

**Celebrity**	**Male **	**Female **	**%**
Michael Jackson (pop star)	247	242	97.8
Nawaz Sharif (politician)	238	224	92.4
Salman Khan (actor)	240	214	90.8
Rana Naved (player)	232	217	89.8
Aamir Khan (actor)	224	213	87.4
Shoaib Akhtar (player)	201	194	79.0

## DISCUSSION

Plastic surgery is overlapped by many other specialties unlike other surgical disciplines which have more clearly defines areas and restrict themselves to certain anatomical boundaries.^1^ Because of the breadth of plastic surgery and the diversity of the procedures, much information needs to be spread in the society. According to American Society for Aesthetic Plastic Surgery, nearly 8.3 million cosmetic procedures were performed in 2003.^8^ This represents an increase of 299% since 1997.^8^ The marketing of cosmetic surgery and related services to the patients is becoming a major objective.^1^ As a result, the public is becoming more aware of the role of the plastic surgery. The specialty of plastic surgery in not a very new one. The development of plastic surgery as a specialty has resulted in a significant positive impact on health care. 

The present study is of the first of its kind in Pakistan assessing the perceptions of college students about plastic surgery. Majority of the students did not see a plastic surgery procedure and only 5.6% of the respondents had a personal experience of having a plastic surgery procedure. Surprisingly, more than half of the students (57.4%) showed the intention to have a plastic surgery procedure sometimes in their life. Most of the information about plastic surgery was received by internet (88%). The different kind of magazines and advertisements resulted in attracting the students (85.2%). TV shows and advertisement attracted 34.2% students. All these advertisements resulted in friends-gup shup and surprisingly in 62.8% students, these discussions were the primary source of the information.

Another important point was also noted that general practitioners gave some kind of information in only 2.6% of the students. This indicates poor knowledge of the specialty among the general public and even in the doctors. This also warrants the extension of the similar study among doctors. Thousands of hours are spent in plastic surgery training; none of the time is dedicated to community relations and public information.^1^ We have learned how to provide a service but not how to sell the product.

A similar study conducted in the university students in USA indicated that 48% of students would consider plastic surgery operations as compared to 57.4% in our study.^6^ Only 14.6% of students showed their willingness to undergo such surgery in the study by Adeyemo *et al.*^5^ The less number of the students showing willingness may be due to the fact that it was conducted in third world county (Nigeria). Nigeria has a literacy rate of 72.0% as compared to Pakistan 54.2%.^9^^,^^10^ The most important difference between these two studies is that our study was conducted among students whereas the study by Adeyemo *et al.* was conducted in the professionals in banking industry and civil services.^5^

The most common plastic surgery conditions were related to hair (89.9%) followed by face scars (88.0%) and nose (83.4%). But in the study by Agarwal *et al.*, burns were the most common condition (20.4%) as compared to 73.4% in our study.^1^ The most frequently named procedures was liposuction (88.2%) followed by hair transplant surgery (84.4%) whereas liposuction was named in 53% in the study by Adeyemo *et al.*^6^

We have included a very important factor in our study which was not included in other studies^1^^,^^6^^,^^7^^,^^11^ and that was the ideas about scars after having plastic surgery operations. The majority of students (53.4%) thought the scars would be invisible. One fourth of the students had the misconception that there would not be any scar. Only 1.6% thought that it would be a quite visible scar. 

We also included a question to name five celebrities having some kinds of plastic surgery procedure. The ‘face’ of the plastic surgery, Michael Jackson was at the top of the list (97.8%) followed by former Pakistani Prime Minister, Nawaz Sharif (92.4%). 

This survey suggested that the perceptions of plastic surgery in our society were limited and underestimated the versatility of the specialty. Therefore, we recommend improved liaison with general practitioners and the institutions of public awareness programs. Public awareness programs should be instituted through the print media and on TV and should be done judiciously and with dignity. The limitations of our study may be that the students sample was obtained from the same geographical area and this might underestimate the true nature of the awareness level. Despite the rapid growth of plastic surgery in the last two decades, a large portion of population remains unaware of the spatiality. It is essential to institute programs to educate health care consumers and providers about the plastic surgery. 

## CONFLICT OF INTEREST

The authors declare no conflict of interest.

## Footenote

Please cite this paper as:

Ahmad M, Mohmand H, Ahmad N. Views of College Students on Plastic Surgery. World J Plast Surg 2013;2(2): 104-110.
